# Importance of Organized Efforts

**Published:** 1903-02

**Authors:** George M. Holden

**Affiliations:** Hackettstown, N. J.


### IMPORTANCE OF ORGANIZED EFFORTS.[1]

BY GEORGE M. HOLDEN, D.M.D., HACKETTSTOWN, N. J.

Mr. President and Gentlemen,—Organization is always necessary to bring out the best that is in every profession. Dentistry has much to be grateful for in the large number of well-

[1] Read before the Harvard Dental Alumni Association, June 23, 1902.

organized societies and associations that have contributed and are contributing so much for our profession. In this progressive age nearly every branch of human activity has found the importance and value, if not the absolute necessity, of organized association, and we have reason to feel proud of the position that our profession occupies in this advancement.

If the meeting together in a social way only were to be considered, the benefits obtained would be many; for in this way many ideas are acquired that are helpful in our every-day practice, and invaluable friendships are formed. There is no one who cannot, out of his own individual experience, impart some help to others. Friendships have been formed at dental society meetings that could not be more true. I have in mind one instance that may not be out of place to mention here. It was the testimony of an elderly gentleman who had labored long and faithfully at his profession, and by fortunate investments had accumulated considerable wealth. Misfortune overtook him; the savings of a lifetime were suddenly swept away. His description of the practical comfort and unsolicited assistance he received from friends he had made in dental societies was most convincing of their value. He was comforted, encouraged, and stimulated; he had a taste of something more valuable than wealth; he felt the depth and warmth of true friendship.

The carefully planned meetings include other features that serve as a substitute for some of the advantages of the dental school, or, rather, a continuation of them. We have in these the privilege of hearing papers read and discussed by men who have given especial thought to the subjects presented, and the impressions made upon us when listening to these are deeper and more profitable than when we read them in the publications. We have clinics to demonstrate new ideas, and to teach new methods of performing the ordinary operations, and the practical value of these is often great. We also have exhibits that contain the latest inventions and improvements, arranged in an attractive manner; and these, coming from so many sources, enable us to make comparisons and selections expeditiously.

When we are deprived of the devoted supervision of our professors, instructors, and demonstrators, and have to depend upon ourselves to solve the problems that confront us, we find difficulties unthought of, and we long for their friendly counsel. It is then, perhaps, that we first fully appreciate what they have been to us, and many of us have to seek help elsewhere. There are many good magazines that contain much for our profit, and these are indispensable, but they alone will not suffice; we must unite with others in personal efforts to promote our interests and the interests of our profession.

It is important that we do this as soon as possible after leaving the dental school, that the continuity of our studying may not be broken and that our growth may be stimulated. Probably none of the young men who will leave this place in a few days to commence practice will choose locations that are too far from the meeting-place of some dental society to make joining it impossible or impracticable; and, as it is a duty as well as a privilege to do all that we can to maintain these institutions whose objects are so worthy, it is earnestly hoped that they will avail themselves of their advantages at the first opportunity. Many have neglected this at first, and when urged to join later, express regret that they did not join when they began practising. Some become so well satisfied with their own efforts that they say they cannot see how they could be benefited, and some, by departing from the right course, become ineligible. The last danger can be eliminated here, of course, for we well know that your environment during your course of study here has been such as to inculcate the highest principles.

The attainments of men vary in proportion to their ability and application. Some have to acquire by hard work that which comes comparatively easy to others. Each of us, however, can strive to do the best he can, each of us apply himself to hard work; and we can all improve our ability by faithful application, and all be helped by united effort.

It should not be from entirely selfish motives that we assist in the support of these organizations, but that we may be able to help in maintaining and extending their usefulness and continuing their activity. We know that they were founded on pure principles and have become helpful by the self-sacrificing devotion of men who have the best interests of dentistry at heart.

If we study the history of our dental societies and associations, we find that the brightest lights of our profession have been encouragers and promoters of them. We find that they have given freely of their time and energy in making experiments and investigations, and presented the results of these before them; we find that they recognized their importance as an elevating influence and considered it a privilege to attend their assemblies, aid in their support, and add to the interest of their meetings.

Whether dentists join in united efforts or not, they do not fail to feel the helpful influence of them, and they all profit by them in many ways. The scope of the dental societies is so wide that it embraces nearly every department of our calling. Some suggestions made and ideas explained at conventions have shown demands for improvements and new inventions, that have caused the supply that benefits all.

There is a certain amount of unfortunate indifference in regard to the importance of becoming affiliated with the societies. There are some who attend the conventions, profit by them, have a good time, and yet fail to identify themselves with societies. Some search for imperfections, magnify and criticise them. What organization exists that has not imperfections in it, and that could not be better? It is sufficient for us to know that their principles are good, and that there is enough good in them to make them helpful. It is not necessary to have everything we see and hear appeal to us, but there is always enough good that will, and we can discriminate.

Then there may be a feeling among some that the societies are so large and successful that they do not need additional members. This is a mistake, for there is inspiration in numbers, and the enthusiasm of the young is needed as well as the wisdom of the experienced. New societies are being formed all the time, and these should be encouraged. The field is large and the opportunities for usefulness are numerous and constantly increasing. The societies need the co-operation of every reputable dentist.

We should not simply be satisfied to have our names in the lists of members, but we should exert our influence in their behalf by describing their advantages, testifying to the help they have been to us, and urging our brothers to come with us and partake of the pleasure and profit they provide.

Let us all do everything we can to emphasize the importance of becoming identified with organized efforts to promote the interests of the profession we love.

